# Acute kidney injury can predict in-hospital and long-term mortality in elderly patients undergoing hip fracture surgery

**DOI:** 10.1371/journal.pone.0176259

**Published:** 2017-04-20

**Authors:** Seong Eun Hong, Tae-Young Kim, Je-Hyun Yoo, Jwa-Kyung Kim, Sung Gyun Kim, Hyung Jik Kim, Young Rim Song

**Affiliations:** 1 Department of Internal Medicine, Hallym University Sacred Heart Hospital, Anyang, Korea; 2 Department of Orthopedic Surgery, Hallym University Sacred Heart Hospital, Anyang, Korea; 3 Hallym University Kidney Research Institute, Seoul, Korea; Universidade Estadual Paulista Julio de Mesquita Filho, BRAZIL

## Abstract

**Background:**

Hip fracture is a common health problem in the elderly that is associated with increased mortality. Acute kidney injury (AKI) is a frequent complication in elderly patients undergoing surgery and is associated with the clinical outcome. We evaluated the incidence and risk factors of AKI in elderly patients undergoing hip fracture surgery and the impact of AKI on short- and long-term clinical outcomes.

**Methods:**

We performed a retrospective cohort study of 450 elderly patients who underwent hip fracture surgery between January 2010 and December 2012. We defined AKI according to the Acute Kidney Injury Network (AKIN) criteria and investigated the effect of AKI on the duration of hospital stay and in-hospital and long-term mortality.

**Results:**

Of the 450 patients, 95 (21.1%) developed AKI during hospitalization and 178 (39.6%) died, with a mean follow-up of 3.6 ± 1.0 years. The baseline serum creatinine level, use of angiotensin-converting enzyme inhibitors or angiotensin-II receptor antagonists, red blood cell transfusion volume, and history of coronary artery disease were independent risk factors for AKI. Patients with AKI during hospitalization had significantly longer hospital stays and higher in-hospital and long-term mortality than those without AKI. Multivariate analysis revealed that age, history of coronary artery disease, serum albumin level, and AKI were independent predictors of long-term mortality.

**Conclusions:**

AKI is a frequent complication in elderly patients undergoing hip fracture surgery and is independently associated with increased in-hospital and long-term mortality.

## Introduction

Hip fracture is a major health problem in the elderly that is associated with significantly increased morbidity and mortality [1–8]. The estimated mortality associated with hip fractures is 5~10% within 1 month and 12~37% at 1 year depending on both the pre- and post-fracture health status, which can be compromised by intercurrent infection, malnutrition, performance status, cardiovascular disease, and thromboembolism [5,6,8–11]. The excess mortality following hip fracture is sustained for several years and comorbidities such as cardiovascular disease, infection, chronic obstructive pulmonary disease, and dementia increase hip fracture-related mortality [5,12,13].

Acute kidney injury (AKI) is a common morbidity in the hospitalized elderly and is a frequent complication after hip fracture surgery. Electrolyte imbalance and chronic kidney disease are related to the in-hospital mortality, and preoperative renal dysfunction is associated with long-term mortality in elderly patients with hip fracture [7,12,14–20]. However, few studies have examined the impact of AKI on long-term mortality in elderly patients after hip fracture. The structural and functional changes associated with aging increase the risk of AKI in elderly populations. Age older than 65 years is a risk factor for non-recovery from AKI and even progression to chronic kidney disease [6,16,19,21–23]. The long-term survival of patients with AKI is poor and gets worse with increasing age and even AKI that does not require dialysis is associated with increased mortality [24–27]. Multiple definitions of AKI have led to a great disparity in its reported incidence [14,16,18,21,28]. We used the Acute Kidney Injury Network (AKIN) classification to diagnose AKI during hospitalization and investigated the potential role of AKI as a predictor of long-term mortality following hip fracture surgery.

## Patients and methods

### Study subjects

This was a single-center, retrospective cohort study of 450 patients who underwent hip fracture surgery. The inclusion criteria were age ≥ 65 years, experiencing the hip fracture for the first time, and undergoing hip fracture surgery between January 2010 and December 2012 at Hallym University Sacred Heart Hospital, Anyang, Korea. Patients with previously diagnosed end-stage renal disease on renal replacement therapy, a history of hip disease or fracture, or less than 3 months of follow-up were excluded. During the study period, 524 patients underwent hip fracture surgery. Twenty-one patients were excluded because they were already being treated with chronic dialysis therapy, 14 patients had previous history of hip disease or fracture and 29 patients were lost to follow-up. Standard medical and surgical treatment and follow-up protocols were followed in all patients. Two surgeons performed the hip fracture surgery.

Demographic and biochemical data, and the type and duration of nephrotoxic drugs such as angiotensin-converting enzyme inhibitors (ACE inhibitors), angiotensin-II receptor antagonists (ARBs), diuretics, non-steroidal anti-inflammatory drugs (NSAIDs), and contrast medium during the hospitalization were obtained from the medical records. Blood pressure and heart rate at admission were used as baseline data. Hemoglobin levels and biochemical parameters such as albumin, protein, blood urea nitrogen, and creatinine at admission were defined as baseline blood values. Potential risk factors for AKI were also recorded, including intraoperative parameters such as duration of anesthesia, hemodynamic parameters, and urine output. Comorbidities such as diabetes, hypertension, and a history of coronary artery disease (CAD) or cerebrovascular accident (CVA) were also obtained from the records. Baseline and follow-up creatinine levels were monitored and AKI was defined according to the AKIN classification based on changes in the serum creatinine level. AKI was defined as an absolute increase in the serum creatinine level of more than or equal to 0.3 mg/dL, or a percentage increase in serum creatinine of more than or equal to 50% within the 48 hours. The urine output criteria for AKI were not used in the present study. Apart from serum creatinine level at admission that was defined as the baseline value, follow-up serum creatinine values were routinely available for the first 7 to 14 days after surgery as part of routine care. The absolute changes in serum creatinine were calculated according to the peak and initial baseline creatinine levels. The glomerular filtration rate (GFR) was estimated from the initial serum creatinine level using the Chronic Kidney Disease Epidemiology Collaboration equation (CKD-EPI) for assessing baseline renal function at admission. The CKD-EPI equation was calculated as GFR (mL/min/1.73 m^2^) = 141 × min (serum creatinine/*k*, 1)^α^ × max (serum creatinine/*k*, 1)^−1.209^ × 0.993^Age^ × 1.018 (if female) × 1.159 (if black), where *k* is 0.7 for females and 0.9 for males, α is −0.329 for females and −0.411 for males, min indicates minimum serum creatinine/*k* or 1, and max indicates maximum serum creatinine/*k* or 1 [29].

Primary outcome was long-term, all-cause mortality and secondary outcomes were in-hospital mortality and length of stay. The follow-up time was calculated from the date of surgery to the date of death or the end of the study period (March 2015). The median follow-up time of patients alive at the end of the study was 4.5 years. Information regarding outcomes was obtained through electronic medical records. For patients not followed at our center, information was obtained during telephone interviews. Telephone interviews were conducted with the patients or family members of patients after obtaining verbal consent. The cause of death was identified by medical records or death certificates.

### Ethics statement

This study was approved by the local Institutional Review Board/Ethics Committee of Hallym University Sacred Heart Hospital, and it was conducted in compliance with the Declaration of Helsinki. As this work was a retrospective observational study, we did not obtain informed consents. Personally identifiable information of patients was encrypted and all the analyzed data were anonymized.

### Statistical analysis

Statistical analyses were performed using SPSS for Windows software (ver. 18.0; SPSS Inc., Chicago, IL, USA). Continuous variables are expressed as means ± standard deviation, and categorical variables are reported as numbers and percentages. The difference was analyzed by an independent t-test for continuous variables and χ^2^ test for categorical variables. The odds ratios (ORs) for AKI were determined by crude and multivariate logistic regression analysis. To assess the impact of AKI on in-hospital mortality, we used crude and multivariate binomial logistic regression analysis. The following variables were included in analysis: age, sex, diabetes, hypertension, a previous history of CAD or CVA, and blood levels of albumin, creatinine, and hemoglobin at admission. Survival curves were plotted using the Kaplan–Meier method and were analyzed using log-rank tests. The hazard ratios (HRs) for in-hospital and long-term mortality were determined from crude and multivariate Cox regression analysis, and are presented as the HR and 95% confidence interval (95% CI). *P*-values < 0.05 were deemed to indicate statistical significance.

## Results

### Patients’ characteristics

The study included 450 elderly patients who had undergone hip fracture surgery. The mean age of the patients was 78.9 ± 7.0 years (range: 65–95 years) and 100 (22.2%) were male. There were 153 (34.0%) patients with diabetes, 301 (66.9%) with hypertension, 41 (9.1%) with a history of CAD, and 79 (17.6%) with a past CVA. The mean length of follow-up was 43.0 ± 11.5 months.

During the study period, 178 patients died. Eighteen patients died during hospitalization. Table 1 shows the baseline patient characteristics according to clinical outcome. No significant differences were observed in gender distribution, prevalence of diabetes, hypertension, CVA, the type of surgery, or the use of ACE inhibitors, ARBs, or NSAIDs between the deceased and survived patients. The serum blood urea nitrogen, creatinine, sodium, and potassium levels were also similar in the two groups. However, there were significant differences in age, the prevalence of CAD, use of diuretics, heart rate at admission, T-score for bone mineral density, and red blood cell (RBC) transfusion volume between non-survivors and survivors. The mean ages of the survivors and non-survivors were 76.0 ± 6.0 years and 83.3 ± 6.2 years, respectively (p < 0.001). Compared to survivors, hemoglobin and hematocrit levels were lower among non-survivors, as well as serum albumin and protein levels. The serum creatinine level at admission did not differ according to mortality, while the estimated GFR was significantly lower, and the absolute changes in serum creatinine during hospitalization were higher among the patients who died.

**Table 1:** Baseline characteristics by survival status.

Variables	Total (*n* = 450)	Deceased (*n* = 178)	Survived (*n* = 272)
Age (years)*	78.9 ± 7.0	83.3 ± 6.2	76.0 ± 6.0
Gender, male, *n* (%)	100 (22.2)	42 (23.6)	58 (21.3)
Diabetes, *n* (%)	143(31.9)	52 (29.4)	91 (33.6)
Hypertension, *n* (%)	301(67.0)	111 (62.7)	190 (69.9)
CAD, *n* (%)*	41(9.1)	24 (13.6)	17 (6.3)
CVA, *n* (%)	79(17.6)	33 (18.6)	46 (16.9)
ACEI or ARB, *n* (%)	153(34.2)	58 (33.1)	95 (34.9)
Diuretics, *n* (%)*	159(35.6)	82 (46.9)	77 (28.3)
NSAIDs, *n* (%)	252(56.4)	90 (51.4)	162 (59.6)
MBP (mmHg)	89.9 ± 12.9	90.5 ± 16.1	89.5 ± 10.4
Heart rate (bpm)*	78.5 ± 8.8	79.6 ± 10.5	77.8 ± 7.4
Surgery type, *n* (%)			
Fixation	204 (45.3)	82 (46.1)	122 (44.9)
Replacement	246 (54.7)	96 (53.9)	150 (55.1)
Hemoglobin (g/dL)*	11.3 ± 1.6	11.0 ± 1.6	11.5 ± 1.5
Hematocrit (%)*	34.1 ± 5.5	33.2 ± 4.6	34.7 ± 6.0
BUN (mg/dL)	20.5 ± 10.8	21.8 ± 13.9	19.7 ± 8.1
Creatinine (mg/dL)	0.9 ± 1.1	1.0 ± 1.2	0.9 ± 1.0
eGFR (ml/min/1.73m2)*	74.8 ± 37.5	64.2 ± 35.9	81.6 ± 97.1
Protein (g/dL)*	6.5 ± 0.7	6.4 ± 0.7	6.6 ± 0.7
Albumin (g/dL)*	3.7 ± 0.4	3.5 ± 0.4	3.8 ± 0.4
Bone mineral density score*	-3.6 ± 1.1	-3.8 ± 1.1	-3.5 ± 1.1
RBC transfusion volume (units)*	3.2 ± 2.4	3.6 ± 3.0	2.9 ± 2.0
Duration of surgery (min)	174.5 ± 52.8	171.8 ± 51.6	176.1 ± 54.0
Urine output during surgery (ml/hour)	163.2 ± 218.5	172.4 ± 215.7	157.4 ± 220.5
Absolute change in Creatinine*	0.2 ± 0.3	0.2 ± 0.4	0.1 ± 0.2
AKI, *n* (%)*	78 (17.3)	42 (23.6)	36 (13.2)

Continuous variables are expressed as mean ± standard deviation.

*P <0.05

CAD, coronary artery disease; CVA, cerebrovascular accident; ACEIs, angiotensin converting enzyme inhibitors; ARBs, angiotensin-II receptor antagonists; NSAIDS, non-steroidal anti-inflammatory drugs; MBP, mean blood pressure; BUN, blood urea nitrogen; GFR, glomerular filtration rate; RBC, red blood cell; AKI, acute kidney injury.

### The incidence and risk factors of AKI during hospitalization and its impact on clinical outcomes

In total, 95 (21.1%) patients developed AKI during hospialization according to the AKIN criteria: 74 patients were classified as AKIN stage 1, and 21 as stage 2 or 3. No patient progressed to AKI requiring dialysis. Of the 95 patients, 29 were diagnosed with AKI before surgery. The absolute change in serum creatinine was 0.61 ± 0.46 mg/dL in the AKI group and 0.06 ± 0.11 mg/dL in the non-AKI group. Age, gender, diabetes, hypertension, and the use of diuretics or NSAIDs did not affect the occurrence of AKI. Patients with AKI had a higher prevalence of CAD and CVA, and received ARBs or ACE inhibitors more frequently than those without AKI. Serum blood urea nitrogen and creatinine levels were significantly higher, and the serum albumin level was lower among patients with AKI. Although the preoperative hemoglobin and hematocrit levels did not affect the occurrence of AKI, there was a significant difference in the volume of RBC transfusion between the two groups. Intraoperative hemodynamic parameters (blood pressure and heart rate) and urinary output were similar between patients with and without AKI, but the duration of anesthesia was significantly longer in patients with AKI (Table 2). Univariate logistic regression analysis revealed that baseline serum creatinine and albumin levels, use of ACE inhibitors or ARBs, history of CAD, duration of anesthesia, and RBC transfusion volume were significantly associated with the occurrence of AKI. However, only the baseline serum creatinine (OR = 1.31, 95% CI: 1.04–1.63, *p* = 0.013), use of ACE inhibitors or ARBs (OR = 2.00, 95% CI: 1.18–3.43, *p* = 0.011), RBC transfusion volume (OR 1.12, 95% CI 1.02–1.23, *p* = 0.027), and history of CAD (OR = 2.22, 95% CI: 1.1–4.52, *p* = 0.029) were significant risk factors for AKI after adjusting for age, gender, diabetes, hypertension, history of CAD or CVA, use of ACE inhibitors or ARBs, and baseline serum creatinine.

**Table 2:** Patients’ characteristics and clinical outcomes according to acute kidney injury.

Variables	AKI (*n* = 95)	Non-AKI (*n* = 355)	*p* value
Age (years)	79.3 ± 6.7	78.8 ± 7.1	0.597
Gender, male, *n* (%)	27 (28.4)	73 (20.6)	0.102
Hypertension, *n* (%)	70 (74.5)	231 (65.1)	0.085
Diabetes, n (%)	37 (39.4)	106 (29.9)	0.082
CAD, *n* (%)	17 (18.1)	24 (6.8)	0.001
CVA, *n* (%)	23 (24.5)	56 (15.8)	0.049
ACEI or ARB, *n* (%)	47 (50.5)	106 (29.9)	0.001
Diuretics, *n* (%)	41 (44.1)	118 (33.3)	0.054
NSAIDs, *n* (%)	46 (49.5)	206 (58.2)	0.131
Systolic BP (mmHg)	124.1 ± 18.1	122.2 ± 14.7	0.300
Diastolic BP (mmHg)	75.7 ± 10.9	74.1 ± 9.1	0.143
Hemoglobin (g/dL)	11.1 ± 1.6	11.4 ± 1.6	0.092
Hematocrit (%)	33.8 ± 8.3	34.2 ± 4.7	0.590
BUN (mg/dL)	24.4 ± 12.7	19.6 ± 10.3	0.001
Creatinine (mg/dL)			
Initial	1.3 ± 1.7	0.8 ± 0.8	0.011
Peak	1.8 ± 1.7	0.8 ± 0.8	<0.001
eGFR	63.9 ± 42.4	77.7 ± 35.6	0.004
Protein (g/L)	64 ± 0.7	6.5 ± 0.7	0.294
Albumin (g/L)	3.6 ± 0.5	3.7 ± 0.4	0.027
Surgery type, *n* (%)			0.829
Fixation	44 (46.3)	160 (45.1)	
Replacement	51 (53.7)	195 (54.9)	
Intraoperative characteristics			
Duration of anesthesia (min)	188.9 ± 80.1	170.7 ± 42.3	0.039
Mean systolic BP (mmHg)	107.1 ± 22.7	105.6 ± 21.0	0.594
Mean diastolic BP (mmHg)	51.1 ± 9.1	50.0 ± 11.1	0.448
Urinary output (mL/hour)	130.8 ± 178.8	171.7 ± 227.1	0.110
RBC transfusion volume(units)	3.9 ± 3.6	3.0 ± 2.0	0.017
Length of hospital stay (days)	34.7 ± 19.5	26.4 ± 35.1	0.027
In-hospital mortality, *n* (%)	10 (10.5)	8 (2.3)	<0.001
All-cause mortality, *n* (%)	47 (49.5)	131 (36.9)	0.026

Continuous variables are expressed as mean ± standard deviation.

AKI, acute kidney injury; CAD, coronary artery disease; CVA, cerebrovascular accident; ACEIs, angiotensin converting enzyme inhibitors; ARBs, angiotensin-II receptor antagonists; NSAIDS, non-steroidal anti-inflammatory drugs; BP, blood pressure; BUN, blood urea nitrogen; GFR, glomerular filtration rate; RBC, red blood cell.

Interestingly, AKI significantly affected the short- and long-term clinical outcomes. Patients with AKI had longer hospital stays (34.7 ± 19.5 *vs*. 26.4 ± 35.1 days, respectively, *p* = 0.017) and higher in-hospital (10.5% *vs*. 2.3%, respectively, *p*<0.001) and long-term (49.5% *vs*. 36.9%, respectively, *p* = 0.026) mortality.

### Predictors of in-hospital mortality

In our cohort, 18 patients died during hospitalization: 8 from infection, 6 from cardiac failure, 2 from stroke, and 2 from respiratory failure. Univariate logistic regression analysis showed that female, baseline serum albumin, AKI and use of diuretics were significantly associated with in-hospital mortality. However, female (OR 3.13, 95% CI: 1.04–9.36, *p* = 0.042), albumin (OR 0.20, 95% CI: 0.51–0.77, *p* = 0.019) and AKI (OR 4.61, 95% CI: 1.69–12.63, *p* = 0.003) were significant predictors for in-hospital mortality after adjusting for age, sex, diabetes, hypertension, history of CAD or CVA and baseline blood levels of albumin, hemoglobin and creatinine.

### Predictors of long-term mortality

**Fig 1:**
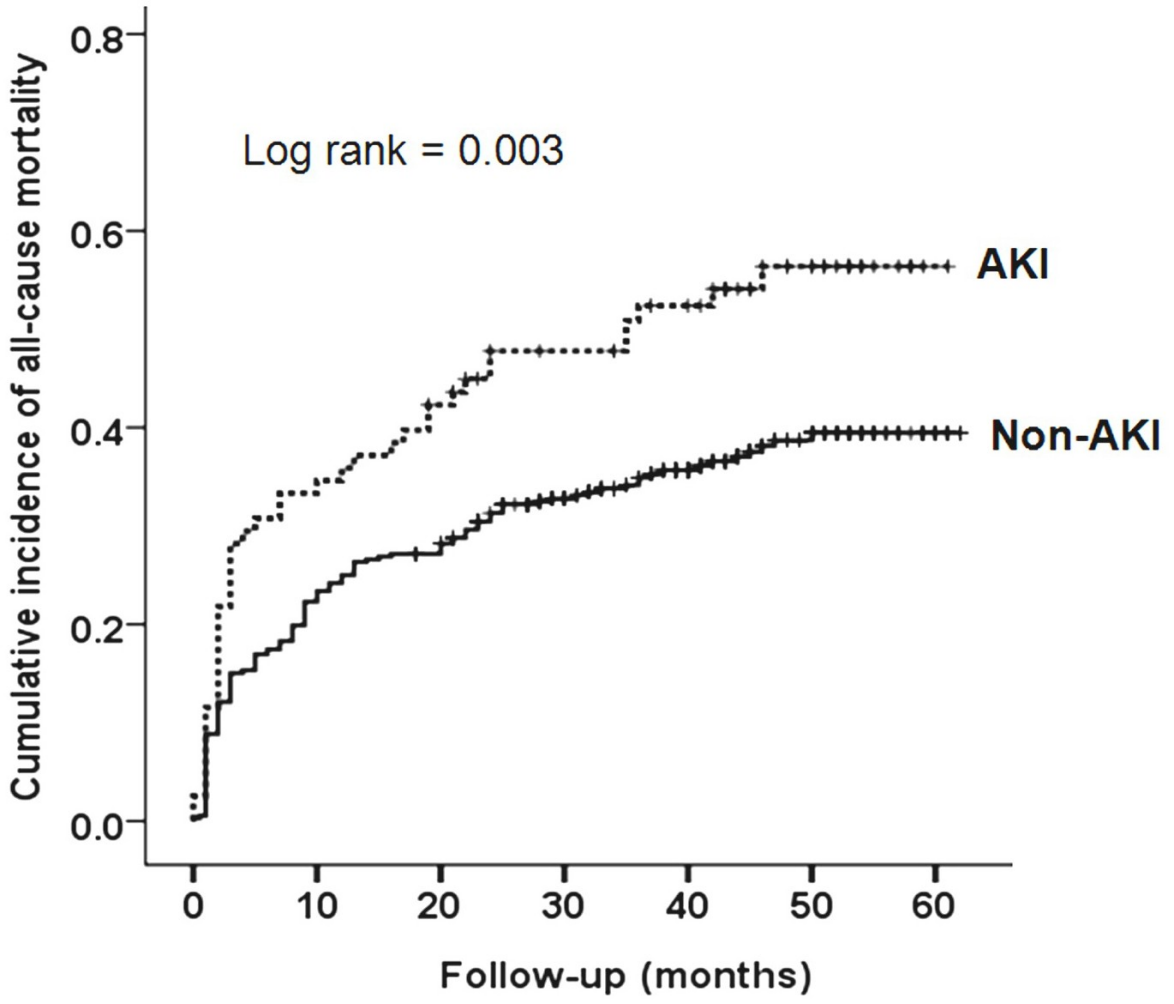
Kaplan-Meier estimates of long-term mortality in elderly patients undergoing hip fracture surgery according to the development of acute kidney injury (AKI).

Fig 1 shows the Kaplan–Meier survival probabilities for all-cause mortality based on AKI. Patients with AKI during hospitalization had significantly increased long-term mortality compared with those without AKI (log rank test, *p* = 0.003). During the study period, 178 patients died: 87 from infection, 37 from cardiovascular disease, 19 from malignancy, 3 from respiratory failure, and 31 from unexplained causes, including sudden death and one case of suicide. The median time to death after discharge was 6.5 months and 78 patients died within the first 3 months after discharge. Overall, the 1-year, 2-year, 3-year and 4-year all-cause death rates were 26.8%, 34.1%, 37.1% and 39.3%, respectively. The clinical predictors of long-term mortality are shown in Table 3. The univariate analysis revealed that age, history of CAD, mean blood pressure and heart rate at admission, hemoglobin and albumin levels at admission, and AKI were significantly associated with all-cause mortality. After adjusting for age, gender, diabetes, hypertension, CAD, CVA, albumin, and hemoglobin, only age (HR = 1.14, 95% CI: 1.11–1.17; p <0.001), history of CAD (HR = 1.93, 95% CI: 1.22–3.04; *p* = 0.005), serum albumin level at admission (HR = 0.44, 95% CI: 0.29–0.68; *p*<0.001), and AKI (HR = 1.63, 95% CI: 1.14–2.31; *p* = 0.007) were significant predictors of long-term, all-cause mortality.

**Table 3:** Cox regression analyses for long-term mortality.

Variables	Univariate			Adjusted		
	HR	95% CI	*p* value	HR	95% CI	*p* value
Age	1.13	1.11–1.16	<0.001	1.14	1.11–1.17	< 0.001
Gender (male)	0.90	0.64–1.27	0.544	0.75	0.52–1.10	0.143
Diabetes	0.89	0.65–1.23	0.486	1.18	0.83–1.68	0.357
Hypertension	0.81	0.60–1.10	0.811	0.78	0.56–1.10	0.155
CAD	1.89	1.23–2.91	0.004	1.93	1.22–3.04	0.005
CVA	1.04	0.71–1.52	0.847	1.24	0.83–1.86	0.298
Mean BP	1.02	1.00–1.03	0.022	1.01	0.99–1.02	0.365
Heart rate	1.02	1.00–1.04	0.032	1.00	0.99–1.02	0.756
Hemoglobin	0.85	0.78–0.94	0.001	1.10	0.86–1.41	0.430
Hematocrit	0.95	0.92–0.98	0.953	0.97	0.90–1.05	0.488
Creatinine	1.07	0.96–1.19	0.222	1.05	0.91–1.21	0.472
Albumin	0.30	0.21–0.43	<0.001	0.44	0.29–0.68	< 0.001
AKI (vs. non-AKI)	1.66	1.18–2.35	0.004	1.63	1.14–2.31	0.007

Multivariate Cox regression analysis was performed after adjusting for age, gender, Diabetes, HTN, CVA, CAD, albumin, hemoglobin, creatinine.

CAD, coronary artery disease; CVA, cerebrovascular accident; BP, blood pressure; AKI, acute kidney injury.

## Discussion

In this study, we found that AKI occurred frequently in elderly patients undergoing hip fracture surgery. Furthermore, AKI not only results in a longer hospital stay and increased in-hospital mortality but also adversely affects long-term survival, even when AKI is less severe and does not require dialysis. To the best of our knowledge, this is the first study to demonstrate the direct effect of AKI on long-term survival in elderly patients undergoing hip fracture surgery. The early diagnosis and prevention of AKI might be very important to improve survival in these patients because hip fracture in the elderly is associated with a marked increase in mortality.

In our cohort, AKI occurred in 21.1% of the patients according to the AKIN criteria. Among AKI patients, most of the patients were classified as AKI stage 1 (77.9%) and no patient progressed to AKI requiring dialysis. In our center, preoperative nephrology consultation is encouraged if estimated GFR is lower than 60 ml/min. Early diagnosis of AKI would be possible, and elderly patients with severe AKI did not undergo surgery. The reported incidence of AKI in elderly patients undergoing hip fracture surgery ranges from 15.3–24.4% according to the definition of AKI and varies with the considered population [2,3,6,8,10,30]. Elderly patients are particularly susceptible to developing AKI. Anatomical and physiological changes in the aging kidney, increased burden of comorbidities, more frequent exposure to medications, and changes in drug metabolism are suspected to contribute to the increased risk of AKI in the elderly [6,8,16,17,19–23]. AKI following hip fracture has multi-factorial causes. Baseline renal function, age, comorbidities, dehydration, nephrotoxic drugs, and malnutrition are documented risk factors for AKI [2,3,8,13,17,21,31]. In our study, the baseline serum level of creatinine, use of ACE inhibitors or ARBs, RBC transfusion volume, and history of CAD were significantly associated with an increased risk of AKI, while the use of NSAIDs was not. This may result from the fact that NSAIDs were used mostly in patients with preserved renal function in our cohort. In fact, the baseline serum creatinine level was significantly lower in patients receiving NSAIDs than in those not receiving them (0.8 ± 0.8 *vs*. 1.1 ± 1.4 mg/dL, respectively, *p* = 0.006), but the absolute change in creatinine was similar in the two groups (1.6 ± 0.3 *vs*. 1.9 ± 0.3 mg/dL, respectively, *p* = 0.291). Although the long-term use of NSAIDs is a well-known risk factor for renal and gastrointestinal complications in elderly patients, they might be relatively safe for short-term use in patients with preserved renal function.

Studies show that AKI is associated with the duration of hospitalization, progression to chronic kidney disease, functional status in daily living, and mortality risk in various populations [6,8,15,16,18,19]. Short-term effects of AKI on clinical outcomes in patients with hip fractures have been demonstrated in several studies, while the long-term impact of AKI on survival has not been described well in these patients. We confirmed that AKI after hip fracture surgery is associated with increased hospital stay and is a major risk factor for in-hospital mortality (OR = 4.61, 95% CI: 1.69–12.63, *p* = 0.003). The main causes of the hospital death were infections and cardiovascular events. AKI may be accompanied by acute liver, lung, and other organ damages. However, our retrospective study cannot demonstrate a causal relationship between AKI and outcomes. A relationship between baseline serum urea and long-term mortality has been reported in patients with hip fracture [4,9]. Excess mortality in patients following hip fracture is documented and the increased mortality risk might persist for several years after the event. Mortality is usually highest during the first year after hip fracture [1,32], as it is confirmed by our study. In our cohort, the rate of all-cause mortality was 26.8, 34.1, 37.1, and 39.3% at 1, 2, 3 and 4 years, respectively. A few studies investigating predictors of mortality following hip fracture have identified age, dementia, malnutrition, and comorbidities [4,7,10,13,20]. Our study suggests that AKI during hospitalization is an important predictor of long-term mortality. Elderly patients with hip fractures at risk of AKI should be identified and appropriate preventive management should be considered to improve survival. We also found a close relationship between albumin and mortality following hip fracture. The close relationship between albumin and mortality has been well documented in various diseases, although serum albumin levels may be affected not only by nutritional status, but also inflammatory and oxidative status [33,34].

This study had several limitations. First, as an observational, retrospective study, there was an inherent design limitation. Second, AKI was defined according to increments in the serum creatinine level only, not in urine output, which is correlated more with renal function [8,18,24,31,35]. Third, we did not consider the type of hip fracture, operative technique, or waiting time for surgery, which might influence outcomes. Finally, because we did not investigate residual renal function after discharge, we cannot conclude that chronic kidney disease after AKI affects the long-term outcomes or whether other factors are associated with mortality.

In summary, AKI is a common complication following hip fracture surgery and baseline renal function, use of ACE inhibitors or ARBs, RBC transfusion volume, and previous CAD were significant risk factors for AKI. The increased mortality following hip fracture surgery persisted for 4 years after the event and AKI was a significant predictor of in-hospital and long-term mortality. However, prospective studies should evaluate the relationship between AKI and clinical outcomes and the benefit of early diagnosis and preventive management of AKI in patients with hip fracture.

## References

[1] Haentjens P, Magaziner J, Colón-Emeric CS, Vanderschueren D, Milisen K, Velkeniers B, et al (2010) Meta-analysis: excess mortality after hip fracture among older women and men. Annals of internal medicine 152: 380–390. 10.7326/0003-4819-152-6-201003160-00008 20231569 PMC3010729

[2] Yun Y-S, Yu J, Kim JH, Kwon KW, Lee HS, Lee YB, et al (2013) Risk Factors and the Clinical Course of Acute Kidney Injury in Patients with a Femoral Fracture. Korean Journal of Medicine 84: 818–826.

[3] Eren Z, Uluçay Ç, Kaspar EÇ, Altıntaş F, Kantarcı G (2012) Acute kidney injury after hip fracture surgery among aging population: Evaluation of incidence and covariates. European Geriatric Medicine 3: 345–348. 10.1177/2151458512473827 PMC3598517 23569709

[4] Seyedi HR, Mahdian M, Khosravi G, Bidgoli MS, Mousavi SG, Razavizadeh MR, et al (2015) Prediction of Mortality in Hip Fracture Patients: Role of Routine Blood Tests. Archives of bone and joint surgery 3: 51 25692170 PMC4322126

[5] Forsen L, Søgaard A, Meyer H, Edna T-H, Kopjar B (1999) Survival after hip fracture: short-and long-term excess mortality according to age and gender. Osteoporosis International 10: 73–78. 10501783 10.1007/s001980050197

[6] Ulucay C, Eren Z, Kaspar EC, Ozler T, Yuksel K, Kantarci G, et al (2013) Risk factors for acute kidney injury after hip fracture surgery in the elderly individuals. Geriatric orthopaedic surgery & rehabilitation: 2151458512473827. 10.1177/2151458512473827 PMC3598517 23569709

[7] Khan SK, Rushton SP, Courtney M, Gray AC, Deehan DJ (2013) Elderly men with renal dysfunction are most at risk for poor outcome after neck of femur fractures. Age and ageing 42: 76–81. 10.1093/ageing/afs152 23034557

[8] Bennet SJ, Berry OM, Goddard J, Keating JF (2010) Acute renal dysfunction following hip fracture. Injury 41: 335–338. 10.1016/j.injury.2009.07.009 19729159

[9] Lewis J, Hassan S, Wenn R, Moran C (2006) Mortality and serum urea and electrolytes on admission for hip fracture patients. Injury 37: 698–704. 10.1016/j.injury.2006.04.121 16815394

[10] Kayatas K, Sahin G, Tepe M, Kaya ZE, Apaydin S, Denmirtunc R (2014) Acute kidney injury in the elderly hospitalized patients. Renal failure 36: 1273–1277. 10.3109/0886022X.2014.934693 24986184

[11] Talsnes O, Hjelmstedt F, Dahl OE, Pripp AH, Reikerås O (2012) Biochemical lung, liver and kidney markers and early death among elderly following hip fracture. Archives of orthopaedic and trauma surgery 132: 1753–1758. 10.1007/s00402-012-1611-7 22996053

[12] Hagino T, Ochiai S, Watanabe Y, Senga S, Saito M, Takayama Y, et al (2013) Hyponatremia at admission is associated with in-hospital death in patients with hip fracture. Archives of orthopaedic and trauma surgery 133: 507–511. 10.1007/s00402-013-1693-x 23411935

[13] Petersen M, Jørgensen H, Hansen K, Duus B (2006) Factors affecting postoperative mortality of patients with displaced femoral neck fracture. Injury 37: 705–711. 10.1016/j.injury.2006.02.046 16765352

[14] Maaravi Y, Bursztyn M, Hammerman-Rozenberg R, Stessman J (2007) Glomerular filtration rate estimation and mortality in an elderly population. Qjm 100: 441–449. 10.1093/qjmed/hcm043 17553810

[15] Chertow GM, Burdick E, Honour M, Bonventre JV, Bates DW (2005) Acute kidney injury, mortality, length of stay, and costs in hospitalized patients. Journal of the American Society of Nephrology 16: 3365–3370. 10.1681/ASN.2004090740 16177006

[16] Maaravi Y, Bursztyn M, Hammerman-Rozenberg R, Cohen A, Stessman J (2006) Moderate renal insufficiency at 70 years predicts mortality. Qjm 99: 97–102. 10.1093/qjmed/hcl002 16407374

[17] Harty J (2014) Prevention and Management of Acute Kidney Injury. The Ulster medical journal 83: 149 25484464 PMC4255835

[18] Macedo E, Malhotra R, Claure-Del Granado R, Fedullo P, Mehta RL (2010) Defining urine output criterion for acute kidney injury in critically ill patients. Nephrology Dialysis Transplantation: gfq332. 10.1093/ndt/gfq332 PMC3108356 20562094

[19] Abdel-Kader K, Palevsky PM (2009) Acute kidney injury in the elderly. Clinics in geriatric medicine 25: 331–358. 10.1016/j.cger.2009.04.001 19765485 PMC2748997

[20] Anderson S, Eldadah B, Halter JB, Hazzard WR, Himmelfarb J, Horne FM, et al (2011) Acute kidney injury in older adults. Journal of the American Society of Nephrology 22: 28–38. 10.1681/ASN.2010090934 21209252

[21] Levey AS, Becker C, Inker LA (2015) Glomerular filtration rate and albuminuria for detection and staging of acute and chronic kidney disease in adults: a systematic review. JAMA 313: 837–846. 10.1001/jama.2015.0602 25710660 PMC4410363

[22] Rosner MH. Acute kidney injury in the elderly; 2009. 10.1016/j.cger.2013.05.001 23849008

[23] Mårtensson J, Bellomo R (2014) Prevention of renal dysfunction in postoperative elderly patients. Current opinion in critical care 20: 451–459. 10.1097/MCC.0000000000000107 24999794

[24] Hobson CE, Yavas S, Segal MS, Schold JD, Tribble CG, Layon AJ, et al (2009) Acute kidney injury is associated with long-term mortality after cardiothoracic surgery. Circulation 119:2444–2453. 10.1161/CIRCULATIONAHA.108.800011 19398670

[25] Coca SG, Yusuf B, Shlipak MG, Garg AX, Parikh CR (2009) Long-term risk of mortality and other adverse outcomes after acute kidney injury: a systematic review and meta-analysis. Ameran Journal of Kidney Disease 53: 961–973. 10.1053/j.ajkd.2008.11.034 PMC2726041 19346042

[26] Bihorac A, Yavas S, Subbiah S, Hobson CE, Schold JD, Gabrielli A, et al (2009) Long-term risk of mortality and acute kidney injury during hospitalization after major surgery. Annals of Surgery 249:851–858. 10.1097/SLA.0b013e3181a40a0b 19387314

[27] Newsome BB, Warnock DG, McClellan WM, Herzog CA, Kiefe CI, Eggers PW, et al (2008) Long-term risk of mortality and end-stage renal disease among the elderly after small increases in serum creatinine level during hospitalization for acute myocardial infarction. Archives of Internal Medicine 168: 609–616. 10.1001/archinte.168.6.609 18362253

[28] Sever MS, Kellum J, Hoste E, Vanholder R (2011) Application of the RIFLE criteria in patients with crush-related acute kidney injury after mass disasters. Nephrology Dialysis Transplantation 26: 515–524. 10.1093/ndt/gfq426 20647191

[29] Michels WM, Grootendorst DC, Verduijn M, Elliott EG, Dekker FW, Krediet RT (2010) Performance of the Cockcroft-Gault, MDRD, and new CKD-EPI formulas in relation to GFR, age, and body size. Clinical Journal of the American Society of Nephrology 5: 1003–1009. 10.2215/CJN.06870909 20299365 PMC2879308

[30] Carpintero P, Caeiro JR, Carpintero R, Morales A, Silva S, Mesa M (2014) Complications of hip fractures: A review. World journal of orthopedics 5: 402 10.5312/wjo.v5.i4.402 25232517 PMC4133447

[31] de Geus HR, Betjes MG, Bakker J (2012) Biomarkers for the prediction of acute kidney injury: a narrative review on current status and future challenges. Clinical kidney journal 5: 102–108. 10.1093/ckj/sfs008 22833807 PMC3341843

[32] Cameron ID, Chen JS, March LM, Simpson JM, Cumming RG, Seibel MJ, et al (2010) Hip fracture causes excess mortality owing to cardiovascular and infectious disease in institutionalizedolder people: a prospective 5-year study. 10.1359/jbmr.091029 19839771

[33] Pioli G, Barone A, Giusti A, Oliveri M, Pizzonia M, Razzano M, et al (2006) Predictors of mortality after hip fracture: results from 1-year follow-up. Aging clinical and experimental research 18: 381–387. 17167302 10.1007/BF03324834

[34] Goldwasser P, Feldman J (1997) Association of serum albumin and mortality risk. Journal of clinical epidemiology 50: 693–703. 9250267 10.1016/s0895-4356(97)00015-2

[35] Vanmassenhove J, Vanholder R, Nagler E, Van Biesen W (2013) Urinary and serum biomarkers for the diagnosis of acute kidney injury: an in-depth review of the literature. Nephrology Dialysis Transplantation 28: 254–273. 10.1093/ndt/gfs380 23115326

